# Fragile X-Associated Tremor/Ataxia Syndrome: Unmet Needs and a Path for the Future

**DOI:** 10.3389/fgene.2018.00100

**Published:** 2018-06-07

**Authors:** Deborah A. Hall, Randi J. Hagerman

**Affiliations:** ^1^Department of Neurological Sciences, Rush Medical Center, Chicago, IL, United States; ^2^MIND Institute, University of California, Davis, Sacramento, CA, United States

**Keywords:** FXTAS, unmet needs, clinical care, consortium, research

## Introduction

It has been estimated that there may be as many as 1,000,000 carriers of the *FMR1* premutation in the United States. A significant portion of these individuals will develop medical problems related to the premutation, including fragile X-associated primary ovarian insufficiency (FXPOI) and fragile X-associated tremor ataxia syndrome (FXTAS). FXTAS is a progressive, neurodegenerative disease that manifests in tremor, ataxia, executive dysfunction, and neuropathy. Patients with FXTAS are identified through known fragile X families or, more recently, by an adult neurologist. Currently, there are only a small number of clinicians and clinics around the world that have a FXTAS focus. This has resulted in delayed diagnosis, inadequate genetic counseling, uncertainty in management of the patient, and a lack of approved medications for treatment. The FXTAS clinical and research community has organized and identified a number of unmet needs with a plan for moving the field forward, in order to best serve this population of patients who are suffering.

## Results

Two organized groups have been pivotal in creating a strategic plan for FXTAS and for a FXTAS consortium: the National Fragile X Foundation FXTAS Task Force and the International FXTAS Consortium. These two groups have convened multiple times over the last 3 years and are composed of clinicians, scientists, researchers, patients, and foundation members. The unmet needs described are a result of these meetings and discussions.

### Preclinical work

It is readily acknowledged that preclinical FXTAS research is critical to advances in the care of patients with FXTAS. Preclinical work is needed to target outcome validation and therapy selection, in addition to testing viable medication candidates. Tactics for this work include: confirming the pathophysiology of the CGG repeat and the causal relationship to phenotype, determining downstream effects and potential neurobiological targets for intervention, and determining the best outcome measures for treatment studies. In addition, validated assays for small molecule screening for treatment compounds and access to larger libraries for screening (i.e., NIH or pharmaceutical companies) are also needed.

### FXTAS repositories

As part of the “Third International Meeting on the Premutation: Basic Mechanisms and Clinical Involvement,” a meeting was held with attendees regarding the establishment of a FXTAS repository. FXTAS researchers have been discussing the need for this collaborative effort for years and such a repository would be a way to unite and stimulate the FXTAS research community. A repository is needed to catalog current resources, which include blood samples, autopsy specimens, fibroblast cell lines, and premutation animal models. A significant hurdle in a central repository is funding limitations. Although individual repositories, exist the cross-institutional nature of specimen collection, can be a substantial administrative burden for the repository organizer. Maintaining a central repository will be expensive and to date, there has not been a funding source identified.

### Outcome measures for FXTAS clinical trials

The failure of early clinical trials in fragile X syndrome have informed the FXTAS research community of the need for validated, pertinent clinical outcome assessments (COAs) for FXTAS clinical trials. Three Phase II clinical trials testing memantine, (Yang et al., 2014, 2016) allopregnanolone, (Wang et al., 2017), and an herbal preparation (NCT02197104) have now been completed in FXTAS and some of these compounds may move into Phase III studies in the next few years. However, the field is not yet prepared to test these medications. The measurement of motor features of FXTAS has been done using the FXTAS Rating Scale, the Scale for the Assessment of Tremor, the International Cerebellar Ataxia Cooperative Scale, and quantitative measures such as event related potentials; none of which have been completely validated, longitudinally or across sites. Quality of life measures have not been tested in large sample sizes and this is vital given the lack of insight into the disease's consequences for the family and the need for patient reported outcomes in future studies. The research community needs to fully understand the natural trajectory of FXTAS, including the cognitive and psychiatric components in both men and women so that clinical trials can be powered adequately for appropriate outcome measures. Biomarkers would be ideal to track physiologic changes with COAs and to date, have not been developed and validated in FXTAS.

### Funding mechanisms for FXTAS researchers

The precarious state of government funding for FXTAS has caused a change in the amount and type of research that is being done. Laboratory closures, an exodus of applications for NIH neuroscience related awards (Hall et al., 2018), and increasing demands on researchers have resulted in a lack of growth of research in the FXTAS arena. FXTAS is a rare disease and as such, NIH review sections may see the significance of the work to have a lower impact than more common neurological diseases in adults. Funding for COAs development can be expensive given the number of patients and clinicians needed for clinimetric testing and validation.

### Identification of FXTAS patients

Over the last 10 years, there has been a steady increase in the identification of patients with FXTAS outside of fragile X research sites and clinics. This fits with the average amount of time needed after the discovery of a new disease to be integrated in standard clinical care or diagnostic workup. The slow increase in identified patients is very likely to impact the needed sample size for larger Phase III clinical trials. Ideally, the presence of a well-advertised Phase III multi-center clinical trial will increase the number of patients that neurologists send for testing and subsequently boost sample sizes. Although fragile X research programs have been somewhat successful in converting premutation carriers discovered from cascade testing into clinical research projects, a more concerted approach to retain and recruit these individuals into clinical trials is needed. Fragile X families are very proactive and may also be needed to facilitate patient identification.

### Education for patients and families

FXTAS clinics that offer symptomatic care to the aged, neurological patients are few in number. In addition, patients with FXTAS who are identified outside of the catchment area of these specialized clinics who have resources will often fly in for care and may not have adequate care in their local communities. The National Fragile X Foundation and local investigators/clinicians have worked to provide online resources to the FXTAS community in the form of patient materials and consensus documents for care providers. As research advances and new patients with FXTAS are identified, especially outside of fragile X families, it will be critical to find avenues to continue to deliver education to these individuals.

## Discussion

There are several solutions that have been proposed and could be developed to address these unmet needs in FXTAS. The establishment of an international FXTAS consortium will be useful for stimulating the international awareness and collaboration of many centers worldwide. It will facilitate communication with telemedicine connections for difficult cases and disseminate advances in the FXTAS field to an international audience that is involved in research. When multi-site projects are proposed and funded, it will facilitate cooperation among researchers including adult neurologists who are identifying, recruiting, and treating patients with FXTAS in large clinical trials. It also can serve as an organizational structure for other collaborative projects including the sharing of tissue samples. Developing and obtaining targeted funding from governmental agencies, foundations, and grateful patients will advance the field and may be the most successful of the strategies. Increased funding could also allow for additional recruitment and retention of trainees and faculty who will move into the FXTAS research pipeline. Continuous updates for practicing neurologists, researchers, patients, and families of education and science related to FXTAS will broaden the population of identified patients and improve quality of life of patients. The FXTAS research community is comprised of many clinicians and scientists who are willing and have a history of working together in a collaborative and collegial manner. This environment will help in reaching the goals needed for the FXTAS population.

## Author contributions

DH: Drafting and revision of the manuscript. RJH: Revision of the manuscript.

### Conflict of interest statement

The authors declare that the research was conducted in the absence of any commercial or financial relationships that could be construed as a potential conflict of interest.
